# Discovery of RGS2-FBXO44 interaction inhibitors using a cell-based NanoBit assay

**DOI:** 10.1016/j.molpha.2025.100030

**Published:** 2025-03-19

**Authors:** Sadikshya Aryal, Cindy Shi Yee Wong, Harrison J. McNabb, Ahmad Junaid, Ryan A. Altman, Benita Sjögren

**Affiliations:** 1 Borch Department of Medicinal Chemistry and Molecular Pharmacology, Purdue University, West Lafayette, Indiana; 2 Department of Pharmaceutical Sciences, University of California, Irvine, Irvine, California

**Keywords:** High-throughput screening, Proteasomal degradation, RGS proteins

## Abstract

Regulators of G protein signaling (RGS) proteins negatively regulate signaling through G protein-coupled receptors, and reduced RGS protein function is involved in numerous pathologies. However, therapeutic intervention is challenging, as RGS proteins lack druggable binding pockets and enzymatic activity. Instead, targeting mechanisms that control RGS protein expression show promise as an alternative. Pharmacological stabilization of RGS2 would be a feasible therapeutic strategy in pathologies associated with reduced RGS2 protein levels, such as hypertension, heart failure, and asthma. RGS2 is rapidly degraded through the ubiquitin-proteasomal system, and we recently identified the E3 ligase that recognizes RGS2. F-box Only Protein 44 (FBXO44) acts as the substrate recognition site for RGS2 in this E3 ligase complex, and we hypothesize that inhibiting the RGS2-FBXO44 interaction will lead to enhanced RGS2 levels. Here, we developed a NanoLuc Binary Technology (NanoBiT) assay that detects the interaction between RGS2 and FBXO44. This assay was used to screen 1600 compounds from the Life Chemicals protein-protein interaction fragment library. We identified a promising hit, denoted compound **10**, that inhibits the RGS2-FBXO44 interaction with a potency of 19.6 *μ*M, through direct binding to RGS2. The resulting increase in RGS2 protein levels is dependent on FBXO44, as siRNA-mediated FBXO44 knockdown attenuates the effect of compound **10**. Altogether, compound **10** represents the first example of a small-molecule inhibitor of the RGS2-FBXO44 interaction and a first step toward the development of molecular probes with a defined mechanism to stabilize RGS2 protein levels.

**Significance Statement:**

This study provides a strategy to identify molecules that selectively inhibit RGS2 protein degradation as well as the first example of a compound with the ability to inhibit RGS2 interaction with the E3 ligase component FBXO44. This study provides proof of concept that a small-molecule RGS2-FBXO44 interaction inhibitor will increase RGS2 protein levels. Future development of compounds with this mechanism of action would be clinically useful in pathologies associated with low RGS2 protein levels, including hypertension, heart failure, and asthma.

## 1 Introduction

Regulators of G protein signaling (RGS) proteins serve as key negative regulators of signaling through G protein-coupled receptors (GPCRs). They reduce the amplitude and duration of GPCR signaling by accelerating GTP hydrolysis, through their canonical GTPase-accelerating protein (GAP) activity, at active G*α* subunits of heterotrimeric G proteins (Ross and Wilkie, 2000; Sjögren et al, 2010; Sjögren, 2017). Although altered RGS protein function has been implicated in numerous diseased states, therapeutic targeting is challenging due to the lack of druggable binding pockets and enzymatic activity. Furthermore, although the development of RGS protein inhibitors that are active in vivo has been somewhat successful (Blazer et al, 2015), there are many cases in which increasing, rather than decreasing, RGS activity would provide clinical benefit. A notable example is represented by RGS2, a member of the R4 subfamily of RGS proteins, which displays unique selectivity as a GAP for G*α*_q_ over other G*α* subtypes (Heximer et al, 1997). Loss of RGS2 has been implicated as a contributing factor in cardiovascular diseases, such as hypertension and heart failure, as RGS2^−/−^ mice display a hypertensive phenotype and prolonged responses to vasoconstrictors acting through Gq-coupled GPCRs, such as angiotensin II type 1 receptor (Heximer et al, 2003). These mice are also more vulnerable to cardiac injury; stressors, such as thoracic aortic banding, induce augmented cardiac hypertrophy (Takimoto et al, 2009). These, and many other, studies provide support for the potential for enhancing RGS2 function to alleviate cardiovascular deficiencies (Tsang et al, 2010). We previously showed that pharmacologically increasing RGS2 protein levels inhibits Gq-mediated signaling and is cardioprotective in a mouse model of cardiac injury, suggesting that enhanced RGS2 protein levels correlate with increased function (Sjögren et al, 2012; Sjögren et al, 2016). Therefore, targeting mechanisms that regulate RGS2 protein levels should be a feasible strategy to increase RGS2 function.

RGS2 is rapidly degraded through the ubiquitin-proteasomal system (UPS; Bodenstein et al, 2007). In the UPS, a K48-linked chain of ubiquitin molecules is covalently linked to the substrate through the cascade of enzymatic reactions (facilitated by E1 ubiquitin-activating enzyme, E2 ubiquitin-conjugating enzyme, and E3 ligases). The ubiquitinated substrate is then recognized and degraded by the 26S proteasomal complex (Hershko and Ciechanover, 1998). The large superfamily of E3 ligases provides substrate selectivity within the UPS, and inhibiting the interaction between a substrate and its cognate E3 ligase provides a selective strategy to avoid degradation and maintain higher protein levels. We identified an E3 ligase that recognizes RGS2 (Sjögren et al, 2015), consisting of Cullin 4B (CUL4B), DNA Damage-Binding Protein 1 (DDB1), and F-box Only Protein 44 (FBXO44). FBXO44 acts as the substrate recognition component and binds RGS2 through its C-terminal F-box-associated domain (Sjögren et al, 2015). We recently established that RGS2 binds to FBXO44 through a stretch of residues near the N-terminus (McNabb et al, 2020), and removal of this region decreases the association with FBXO44 and protects RGS2 from degradation. Therefore, we hypothesize that a small-molecule RGS2-FBXO44 protein-protein interaction (PPI) inhibitor will inhibit RGS2 degradation and consequently increase RGS2 protein levels and activity.

In this study, we developed a NanoLuc Binary Technology (NanoBiT) assay that was used to screen for small-molecule inhibitors of the interaction between RGS2 and FBXO44. We report the identification of a compound that inhibits the interaction in cells. Future development of this, or other, compounds could serve as possible leads for the development of drugs in pathologies associated with reduced RGS2 protein levels. In addition, this assay can also be used to investigate mechanisms that regulate the RGS2-FBXO44 interaction in a high-throughput manner.

## 2 Materials and methods

### 2.1 Materials

All chemicals were purchased from Millipore Sigma unless otherwise stated.

*DNA Constructs and siRNA****—***pBiT1.3-C[CMV/LgBiT/Hygro], pBiT1.3-N[CMV/LgBiT/Hygro], pBiT2.3-C[CMV/SmBiT/Blast], and pBiT2.3-N[CMV/SmBiT/Blast] were graciously provided by Promega (Madison, WI) prior to commercial availability. These plasmids are now available as the NanoBiT CMV multiple cloning site BiBiT-Ready Vector Set (Promega). FBXO44-SmBiT, FBXO44-LgBiT, SmBiT-FBXO44, LgBiT-FBXO44, RGS2-SmBiT, and RGS2-LgBiT were cloned in-house. Dual Expression BiBiT LgBiT-FBXO44/RGS2-SmBiT was cloned in-house. RGS2-HA and RGS4-HA (in pcDNA3.1) were previously described (Bodenstein et al, 2007). FLAG-FBXO44 was a kind gift from Kevin Glenn, University of Iowa. Nontargeting siRNA Control Pool 1 (D-001206-13-05) and human FBXO44 (M-019201-01-0005) siGENOME SMARTpool siRNA was obtained from Horizon/Dharmacon.

### 2.2 Cell culture

Cells were maintained in a humidified incubator at 37 °C with 5% CO_2_. Human embryonic kidney 293T (HEK-293T; CRL-3216, ATCC) cells were cultured in Dulbecco’s modified Eagle’s medium (DMEM; Gibco, #11995), supplemented with 10% fetal bovine serum (FBS; Gibco, #16000), and 100 U/mL penicillin with 100 *μ*g/mL streptomycin (Gibco, #15140). Stable cell lines were maintained in the same media supplemented with 2 *μ*g/mL blasticidin S HCl (Gibco, #A11139-03). The development of stable HEK-293 cell lines expressing RGS2-ProLabel has been previously described (Sjögren et al, 2012). Media for stable cell lines was replaced every other day. Cells were tested for mycoplasma every 6 months using the MycoAlert mycoplasma detection kit (Lonza, #LT07-318).

### 2.3 Transfections

HEK-293T cells were transfected with DNA plasmids using either Lipofectamine 2000 (#11668), Lipofectamine 3000 (#L3000), and siRNA using Lipofectamine RNAiMAX (#13778; Invitrogen), according to the manufacturer’s instructions, under reduced serum conditions. For transient transfection experiments, assays were performed 24 hours after transfection.

### 2.4 Stable cell line development

Cells were plated in a 6-well plate, and transfection was performed as described above. Media was replaced with DMEM, 10% FBS after 24 hours. Antibiotic selection using blasticidin (Gibco, #A11139-03) was initiated 48 hours after transfection at 3 concentrations (2, 6, and 10 *μ*g/mL). Media was replaced every 2 days, and the cells were monitored visually via a brightfield microscope for growth. To isolate single clones, the cells were harvested by trypsination, pelleted, and resuspended in phosphate-buffered saline (PBS; Gibco, #2509235) supplemented with 0.1% bovine serum albumin (BSA). Propidium iodide (Invitrogen, #BMS500PI) was added to a final concentration of 1 *μ*g/mL to isolate viable cells. One live cell/well in 96-well plates was plated by flow cytometry at Purdue University’s Flow Cytometry and Cell Separation Facility. Wells were monitored and expanded as needed. Single clones were tested for luminescence signal as described below.

### 2.5 Cell viability assay

The cells were plated in DMEM without phenol red (Gibco, #21063), 0.1% BSA in a white, tissue culture-treated 384-well plate (Perkin Elmer) at 15,000 cells/well, unless indicated otherwise, and allowed to attach. The plate was centrifuged at 212 × *g* for 30 seconds to aid in even cell adherence. Compound treatments were initiated 4–5 hours after plating. Following indicated treatment times, media was removed from the plate using BioTek ELx405 plate washer. Additionally, 10 *μ*L of Hank’s Balanced Salt solution (HBSS) with 0.1% BSA was added to each well, followed by 5 *μ*L of glycyl-phenylalanyl-aminofluorocoumarin (GF-AFC; MP Biomedicals) diluted 1:1000 in 100 mM HEPES. Plates were then centrifuged at 212 × *g* for 30 seconds and then incubated at 37 °C for 30 minutes. Fluorescence was detected on a Synergy Neo2 multimode plate reader (BioTek/Agilent).

### 2.6 NanoBit assay

Nano-Glo Live Cell Substrate (LCS) was diluted 1:50 in HBSS to form a 2× stock, and then, 15 *μ*L was added to each well. Plates were shaken on an orbital shaker at 500 rpm for 5 minutes protected from light and then spun down at 1000 rpm for 30 seconds. Plates were incubated at room temperature for 20 minutes, and then, luminescence was detected on a Synergy Neo2 multimode plate reader.

### 2.7 PathHunter ProLabel assay

The PathHunter ProLabel assay was run as described previously (Sjögren et al, 2012; Raveh et al, 2014; Sjögren et al, 2015). Briefly, HEK-293T-RGS2-PL cells were plated at 10,000 cells per well in a white, tissue culture-treated 384-well plate (Perkin Elmer) in DMEM without phenol red (#21063; Gibco) and 0.1% BSA. The cells were allowed to attach for 4 hours, followed by treatment with DMSO, 10 *μ*M MG-132, or 100 *μ*M of compounds. Prior to the detection of RGS2 protein levels by the ProLabel assay, a fluorescent viability assay was performed in the same well, as described above. PathHunter ProLabel reagents (DiscoveRx) were prepared according to the manufacturer’s directions and added to the plate, followed by incubation at room temperature for 1 hour. Luminescence corresponding to RGS2-PL protein levels was detected using a Synergy Neo2 multimode plate reader.

### 2.8 Preparation of cell lysates

The cells were harvested and lysed in a radioimmunoprecipitation assay (RIPA) buffer containing protease inhibitors (50 mM Tris-HCl [pH 7.4], 150 mM NaCl, 0.25% [wt/vol] deoxycholate, 1 mM EDTA, 1% NP-40) supplemented with complete Protease Inhibitor Cocktail EDTA-free (Roche, Indianapolis, IN). Lysates were sonicated for 10 minutes at 4 °C and centrifuged at 500 × *g* for 5 minutes. The supernatant was collected and used for downstream applications. Total protein concentration was determined using the Pierce BCA Protein Assay Kit (Thermo Scientific), and protein concentrations were adjusted using SDS sample buffer (Li-Cor Biosciences).

### 2.9 Coimmunoprecipitation (co-IP)

The cells were harvested in RIPA buffer containing protease inhibitors. The cells were then lysed by incubating on ice with occasional vortexing for 30 minutes. The cell lysates were centrifuged at 9600 × *g* for 10 minutes, and the supernatants were precleared by incubating with 40 *μ*L prewashed protein A agarose beads (Roche) for 1 hour with slow rotation at 4 °C. The samples were then centrifuged at 10,000 rpm for 1 minute, and the supernatant was used to determine total protein concentration with the Pierce BCA Protein Assay Kit (Thermo Scientific). In total, 500 *μ*g of total protein was used for each reaction. The samples were incubated with 100 μM compound **10** for 30 minutes with rotation at 4 °C. An equal amount of DMSO was used as a vehicle. Additionally, 20 *μ*L was removed from each sample (input); 3 *μ*L of rabbit anti-HA antibody (H6908; Sigma Aldrich) and 40*μ*L of protein A agarose beads (Roche) were added to each sample prior to incubation on rotator at 4 °C overnight. The samples were centrifuged at 9600 × *g* for 5 minutes at 4 °C, and the supernatant was removed. The samples were washed once with 1 mL RIPA buffer and then 3× with 1 mL PBS. Bound proteins were eluted by heating the samples at 95 °C in 30 *μ*L of SDS sample buffer for 10 minutes. The samples were centrifuged at 10,000 rpm for 5 minutes at 4 °C, and the supernatants were subjected to SDS-PAGE and western blot analysis.

### 2.10 Cellular thermal shift assay (CETSA)

The cells in 100 mm dishes were harvested by trypsination in reduced serum media and transferred to 15 mL conical tubes. The cells were centrifuged at 300 × *g* for 3 minutes. Media was removed, and the cell pellet was washed with 10 mL of PBS. The cells were then resuspended in 500 *μ*L of PBS and aliquoted into polymerase chain reaction (PCR) tubes. The cells were then heated in a PCR gradient (40–60 °C) for 3 minutes and then transferred to a 25 °C PCR block for 3 minutes. Following this, cells were immediately flash-frozen using liquid nitrogen. The cells were freeze-thawed 3 times to lyse the cells and then spun down at 20,000 × *g* for 20 minutes. The supernatant was then added to sample buffer and heated for 5 minutes at 95 °C before SDS-PAGE and western blot analysis.

### 2.11 SDS-PAGE and western blot

Equal amounts of proteins were loaded onto a 12% SDS-PAGE gel and resolved at 160 V, 0.4 A for 1 hour. Proteins were transferred to an Immobilon-P PVDF membrane (EMD Millipore; IPVH00010) for 2 hours at 160 V, 0.4 A. Following protein transfer, the membrane was blocked at room temperature for 1 hour with Intercept PBS Blocking buffer (Li-Cor, 927-70001) and then incubated in primary antibodies (rabbit anti-HA (1:1000; H6908; Sigma Aldrich), rabbit anti-*β*-actin (1:1000; A2066; Sigma Aldrich), mouse anti-FLAG (1:1000; F1804; Sigma Aldrich), rat anti-HA (1:1000; ROAHAHA; Roche), rabbit anti-FBXO44 (1:1000; HPA003363; Sigma Aldrich), or mouse anti-*β*-actin (1:1000; A2228; Sigma Aldrich) for 2 hours at room temperature. Primary antibodies were diluted in Intercept T20 Antibody PBS diluent (Li-Cor, #927-75001). The membrane was subsequently incubated for 1 hour in goat anti-mouse IRDye 680RD (1:15,000; Li-Cor; 926-68070), goat anti-rabbit IRDye 800CW (1:15,000; Li-Cor; 926-32211), and goat anti-rat IRDye 680RD (1:15,000; Li-Cor; 926-68076) secondary antibodies. Following each antibody incubation, the membranes were washed 4 times with PBS, 0.1% Tween-20. The membranes were imaged using the Azure600 imaging system (Azure Biosystems).

### 2.12 Ligand clustering analysis

To cluster small molecules based on structural similarity, we employed Schrödinger software, leveraging a fingerprint similarity approach to assess molecular similarity and group compounds (Schrödinger, 2024). The radial-type fingerprint approach was applied with precision set to 64-bit. For fingerprint calculation, a functional atom typing scheme was applied. Atoms were characterized by functional type, distinguishing between hydrogen (H), carbon (C), halogen grouped as [fluorine, chlorine (F, Cl)] and [bromine, iodine (Br, I)], chalcogens grouped as [nitrogen, oxygen (N, O)] and [sulfur (S)], and all other atom types categorized as “others.” Bonds were further classified according to their hybridization state. The Tanimoto similarity metric was used for calculating molecular similarity, using compound **F5854-3982** as the reference. Based on these similarity scores, clustering was performed using the Single Linkage method, for which the proximity between 2 clusters was defined by the proximity of their nearest objects, following the nearest-neighbor principle.

### 2.13 Statistical analysis

All data were analyzed using GraphPad Prism 10.0 (GraphPad). Dose-response curves were fit using nonlinear regression. Data sets with 2 groups were analyzed using Student *t* test. Data sets with 3 or more groups were analyzed using one-way ANOVA, as indicated in each figure. Groups were compared with Dunnett’s post hoc test for multiple comparisons. All experiments were run at least 3 times unless otherwise indicated in the figure caption. Data are presented as mean ± SD with a *P* value less than .05 considered significant. These studies were aimed at identifying inhibitors of the RGS2-FBXO44 interaction and were exploratory in nature. Experiments were not designed to test a prespecified statistical null hypothesis. Thus, *P* values stated in figures and tables are descriptive.

## 3 Results

### 3.1 Development of an RGS2-FBXO44 NanoBit cell line

**Fig. 1:**
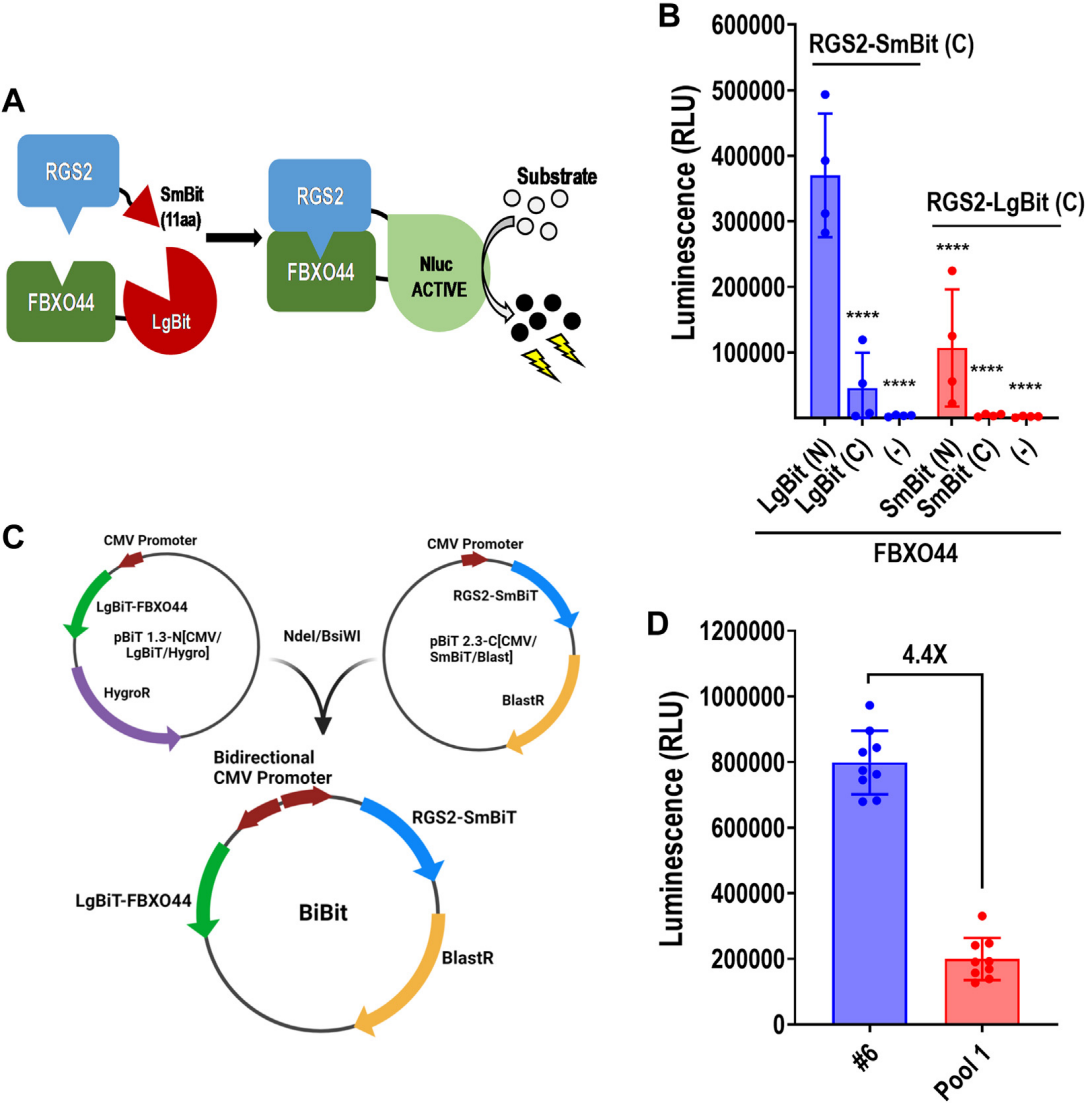
RGS2-FBXO44 NanoBit cell line development. (A) Principle of the NanoBit assay strategy. Nluc complementation is driven by the RGS2-FBXO44 interaction. (B) Each combination of RGS2 and FBXO44 NanoBit construct (RGS2-SmBiT, RGS2-LgBiT, FBXO44-SmBiT, FBXO44-LgBiT, SmBiT-FBXO44, and LgBiT-FBXO44) was tested for luminescence signal intensity. Luminescence was tested using 15,000 cells/well at 2 hours incubation and demonstrated superior signal using RGS2-SmBiT in combination with LgBit-FBXO44. ∗∗∗∗*P* < .0001 using one-way ANOVA with Dunnett’s post hoc test for pairwise comparisons with RGS2-SmBiT/LgBit-FBXO44 (left bar). (C) Schematic showing the creation of BiBiT dual-expression vector with a bidirectional CMV promoter and blasticidin resistance (BlastR) for selection. (D) Raw luminescent signal of Clone 6 (#6) compared with pool 1.

Small molecules with the ability to inhibit the RGS2-FBXO44 interaction would prevent RGS2 protein degradation, enhance RGS2 protein levels, and increase the effect of RGS2 as a negative regulator of G protein signaling. Such molecules could serve as potential leads as therapeutics for pathologies associated with low RGS2 protein levels, such as hypertension and asthma. We opted for a cell-based assay to enable the identification of compounds that would inhibit the RGS2-FBXO44 interaction in a cellular context, in the presence of any posttranslational modifications important for mediating the interaction. We utilized NanoLuc Binary Technology (NanoBiT; Promega), a split luciferase strategy, in which each protein of an interacting pair is tagged with LgBit and SmBit, respectively. The LgBiT (17.6 kDa) subunit has little activity on its own, but spontaneous binding to an 11-amino acid peptide (SmBiT) leads to enzyme complementation that restores NanoLuc Luciferase (Nluc) activity (Dixon et al, 2016; Schwinn et al, 2018). As these 2 components bind with low affinity (∼140 *μ*M), enzyme complementation is driven by the 2 interacting proteins studied (Fig. 1A).

First, we sought to determine the optimal protein tagging configuration for assay development. Because we previously showed that RGS2 interacts with FBXO44 through its N-terminus (McNabb et al, 2020), we only tagged the C-terminus of RGS2 with SmBit and LgBit, respectively. FBXO44 was tagged on both termini, with both fragments. All constructs were cloned and tested for luminescence signal in transiently transfected HEK-293T cells (Fig. 1B). The combination of C-terminally tagged RGS2 (RGS2-Lg/SmBit) with N-terminally tagged FBXO44 (Lg/SmBit-FBXO44) resulted in a far higher luminescence signal than the combinations using C-terminally tagged FBXO44, which agreed with our previous studies using N-terminally tagged FBXO44 for co-IP studies (Sjögren et al, 2015; McNabb et al, 2020). To our surprise, however, there was a large difference in signal depending on which protein was tagged with LgBit and SmBit, respectively. The combination of LgBit-FBXO44 and RGS2-SmBiT delivered a larger signal (3.5-fold) compared with the opposite tagging strategy. Thus, we used this configuration for further assay optimization.

To reduce intra- and inter-assay variability due to transient transfections, we next proceeded to establish a stable cell line utilizing dual-expression promoters present in the RGS2-SmBiT plasmid and inserted LgBit-FBXO44 in the other multiple cloning site. This construct, hereafter referred to as BiBiT (Fig. 1C), provided the benefit that both proteins would be expressed in all transfected cells. In addition, this strategy enabled selection for stable expression using only blasticidin, reducing stress on the cells. Stable pools were tested for NanoBit signal using transient RGS2-SmBiT and LgBiT-FBXO44, transient LgBiT-FBXO44 alone, BiBiT transient transfection, or mock transfection for comparison (Supplemental Fig. 1D). Although pool 2 displayed a significantly higher signal than pool 1 (∼4-fold; Supplemental Fig. 1D), it also displayed much higher variability. Thus, pool 1 was utilized to establish a single-clone cell line. Single clones were established by flow cytometry sorting. Single colonies were expanded and tested for luminescent signal. Our selected clone (#6) displayed a robust signal, 4.4 times above that of the pool (Fig. 1D).

### 3.2 NanoBit assay optimization for high-throughput screening

Our primary assay using the HEK-293T RGS2-FBXO44 NanoBit cell line was extensively optimized to ensure maximum quality for screening. Coupling a fluorescent cell viability assay with our screen would allow us to rule out cytotoxic compounds while also hopefully reducing outliers from pipetting errors while plating cells. Thus, we multiplexed our NanoBit assay with cell viability using a cell-permeable fluorogenic protease substrate (glycyl-phenylalanyl-aminofluorocoumarin; GF-AFC; Niles et al, 2007), which can be combined with a luminescent readout without interfering with the luciferase signal. We previously utilized a similar multiplexing strategy in several screens (Sjögren et al, 2012; Raveh et al, 2014; Sjögren et al, 2015). We assessed and optimized treatment conditions (time, temperature, and reagent volume), cell density, buffer composition, and DMSO tolerance. In addition to the supplied proprietary LCS buffer (Promega), we also tested DMEM, 0.1% BSA, PBS, and HBSS (Supplemental Fig. 1A–C). Luminescent signals using either PBS, HBSS, or LCS buffer were comparable, and we opted to utilize HBSS moving forward. The luminescent signal was stable up to 45 minutes after reagent addition, and we chose to run subsequent assays using 20 minutes of incubation time (Supplemental Fig. 1E). We also observed no effect on either viability or luminescence signal at DMSO concentrations <1% (Data not shown).

**Fig. 2:**
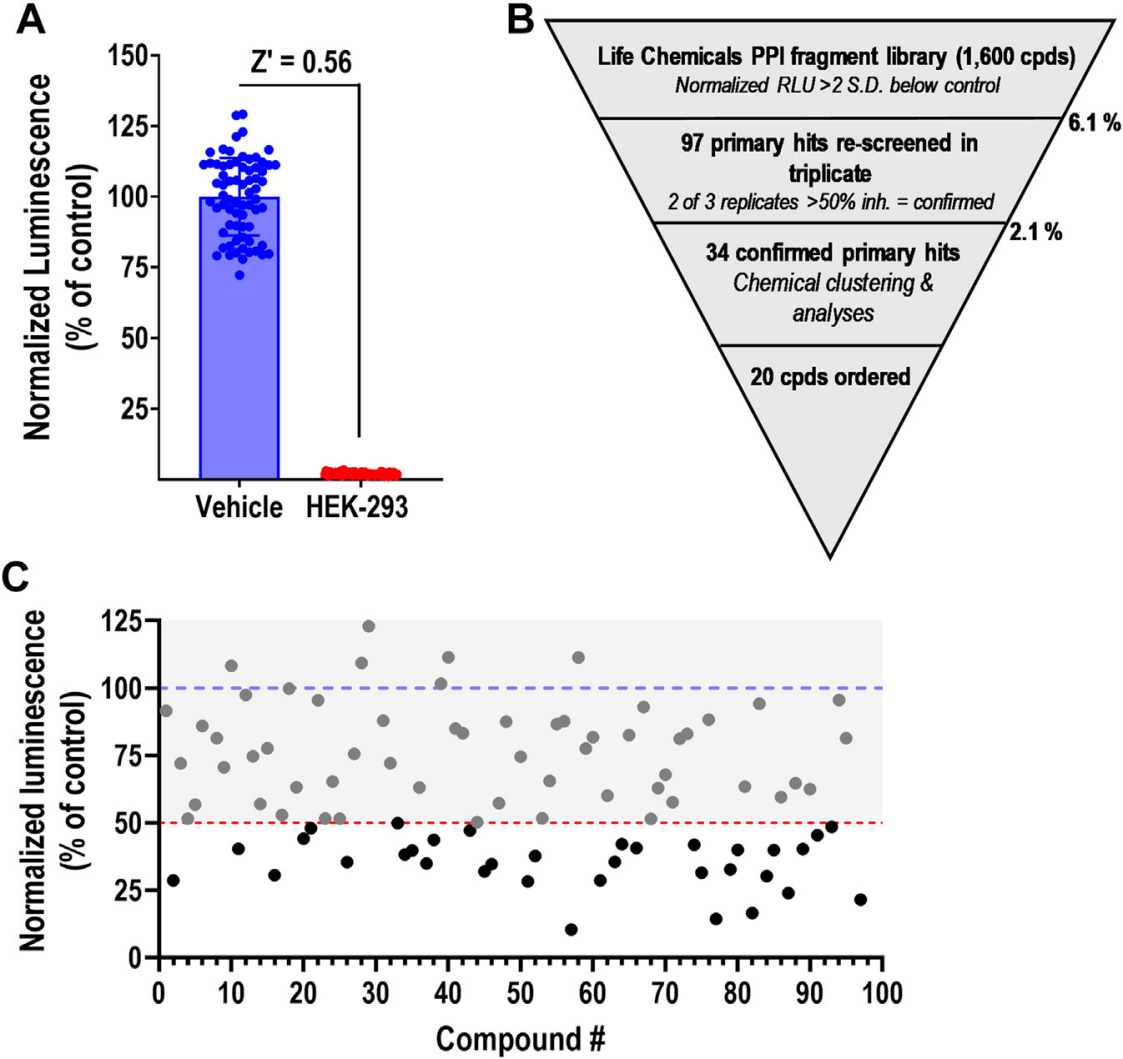
High-throughput screen to identify RGS2-FBXO44 PPI inhibitors. (A) Representation of the Z-factor obtained in the primary screen, as determined by comparing the single clone (#6) RGS2-SmBiT/LgBit-FBXO44 cell line to nontransfected HEK-293T cells. Luminescence was normalized to viability (RLU/RFU) and the average signal in #6 was set to 100%. Z’ was calculated to be 0.56. *N* = 70 for each condition. (B) Screening funnel with hit rates for primary screen and hit confirmation. (C) Scatter plot for hit confirmation. Data presented as normalized luminescence (RLU/RFU) and expressed as percentage of nontreated cells. Blue line represents average response in control cells; Red dotted line represents 50% inhibition of NanoBit signal; Confirmed hits are highlighted in black. RLU, relative luminescence units.

Our final assay demonstrated robust quality, as measured using the Z-factor >0.5 (Zhang et al, 1999), as determined by comparing the response in our single-clone cell line to nontransfected HEK-293T cells (Fig. 2A; Z’ = 0.56 vs nontransfected cells). Although not an optimal negative control, without a preexisting small-molecule inhibitor (which we aimed to identify here), it was the best available option.

### 3.3 Primary screen and hit confirmation

To identify inhibitors of the RGS2-FBXO44 interaction, we screened 1600 compounds from the Life Chemicals PPI Fragment library. The compounds within this library intersect both fragment and PPI inhibitor’s chemical spaces. PPI inhibitors are typically larger and more lipophilic than inhibitors of more standard binding sites of most proteins (Silvestre et al, 2013). Thus, PPI fragments have topological polar surface area values higher than more generic fragment-based libraries. Similarly, although common fragment libraries contain compounds smaller than 300 Da, PPI fragments within this library ranged from 250 to 450 Da.

The workflow for the screen is presented in Figure 2B. HEK-293T-RGS2-FBXO44 NanoBit cells were treated for 16–18 hours with compound at 100 *μ*M. This relatively high concentration was chosen because of the challenging nature of our assay (intracellular PPI inhibition). Parental HEK-293T cells were used as a negative control. Compounds exhibiting less than 25% cell toxicity and inhibition of luminescence signal (relative luminescence units) greater than 2 SD below the signal in vehicle-treated cells were considered hits. Based on these criteria, 97 compounds were identified as hits from our primary screen (6.1% initial hit rate). To confirm hits, the same assay was repeated in triplicate, which delivered 34 compounds that reached the threshold for inhibition in at least 2 replicates with <25% cell toxicity (Fig. 2C; Supplemental Table 1), thus rendering a final hit rate of 2.1%.

To further refine the number of compounds for more rigorous characterization, we next clustered the hits and filtered out compounds with unfavorable properties and functional groups (see Materials and methods). This analysis identified 11 distinct clusters, with the clustering strain quantified at 1.181 (Supplemental Table 1). We used Tanimoto similarity scores to assess the structural resemblance among the clusters (Supplemental Table 1 and Supplemental Fig. 2–4). Following the clustering, medicinal chemistry analysis prioritized 20 hits for further follow-up studies based on (1) assessment of favorable characteristics and drug-like properties and (2) limiting the number of compounds acquired from overrepresented clusters.

### 3.4 Validation of RGS2-FBXO44 NanoBit hits

**Fig. 3:**
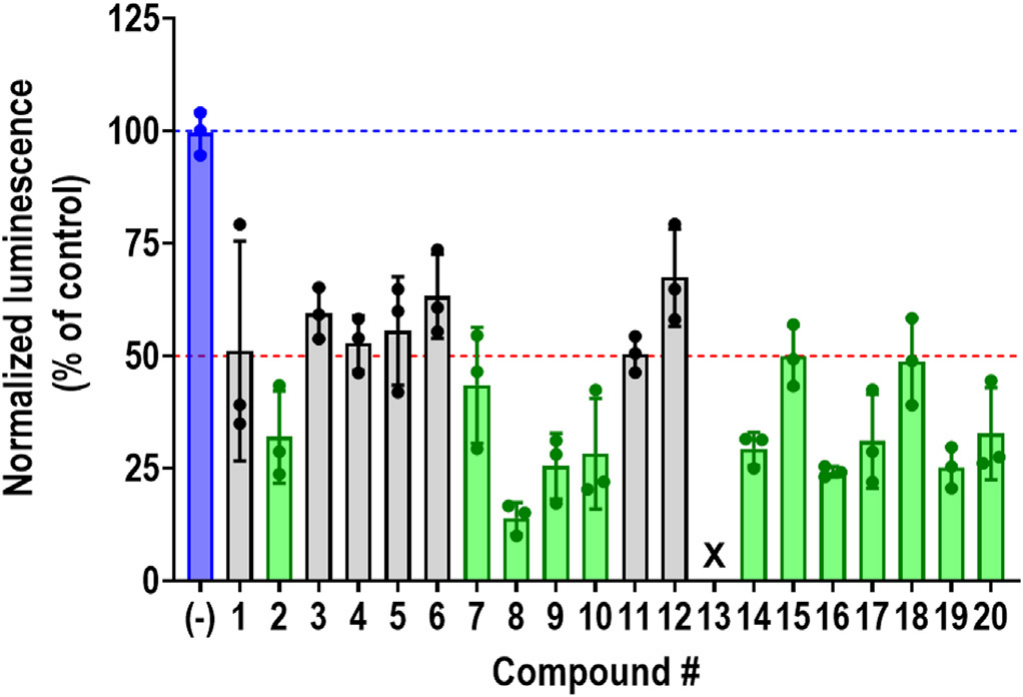
Validation of primary hits. Twenty hits from the primary screen were reordered and assayed for their ability to inhibit the RGS2-FBXO44 NanoBit signal. Luminescence signal was normalized to viability (relative luminescence units/RFU) and expressed as percentage of the signal in nontreated cells. Blue dashed line represents the average signal in the absence of compound. Red dashed line represents 50% inhibition. Results from 3 independent experiments run in triplicate. Although all compounds significantly inhibited RGS2-FBXO44 NanoBit signal (significance levels in Table 1), only about half of them (compounds **2, 7–10** and **14–20;** marked in green) reached the target inhibition of 50% at 100 *μ*M concentration. Compound **13** displayed almost complete cell toxicity and could not be accurately analyzed.

Fresh powders of 20 compounds were purchased and reassayed for their ability to inhibit the RGS2-FBXO44 NanoBit signal, using the same assay paradigm as in the primary screen (Fig. 3; Table 1). When normalized to viability, all 20 compounds significantly inhibited the RGS2-FBXO44 NanoBit signal, with 12 of the compounds reaching the previously defined hit definition of 50% inhibition (compounds **2, 7–10,** and **14–20**). Notably, as fresh powder, compound **13** was toxic at 100 μM (<90%), and the percentage of inhibition could not be accurately defined. Nevertheless, all compounds were confirmed to inhibit the RGS2-FBXO44 NanoBit signal at this high concentration.

**Table 1:** Summary of validation of the 20 reordered confirmed hits Effect of compounds on RGS2-FBXO44 NanoBit signal and cell viability. The results from at least 3 independent experiments run in triplicate. Compound structures and cluster analysis can be found in Supplemental Table 1 and Supplemental Figures 2–4. ∗∗ *P* < .01; ∗∗∗∗ *P* < .0001 using one-way ANOVA with Dunnett’s post hoc test for pairwise comparisons versus untreated cells.

Compound Numbers	CGF ID	Life Chem ID	Cluster	% Inhibition @ 100 *μ*M	Viability (% of Control)	IC_50_ (*μ*M)
1	CGF-0059025	F2075-0589	1	48.9∗∗∗∗	45.8∗∗∗∗	58.0
2	CGF-0220620	F6523-0550	1	68.0∗∗∗∗	101.9	72.2
3	CGF-0219469	F2075-0493	1	40.6∗∗∗∗	54.1∗∗∗∗	78.9
4	CGF-0220386	F6438-1673	2	47.2∗∗∗∗	89.5	38.2
5	CGF-0219442	F2049-0352	3	44.5∗∗∗∗	120.5	150.7
6	CGF-0220231	F5854-3982	4	36.8∗∗∗∗	116.6	25.6
7	CGF-0219368	F1658-1239	4	56.5∗∗∗∗	91.7	42.1
8	CGF-0219375	F1696-0101	4	86.1∗∗∗∗	80.6∗∗∗∗	9.2
9	CGF-0220490	F6464-0765	4	74.5∗∗∗∗	103.6	9.7
10	CGF-0053689	F0838-0022	4	71.7∗∗∗∗	69.3∗∗∗∗	19.6
11	CGF-0219389	F1773-0447	4	49.6∗∗∗∗	139.6	29.0
12	CGF-0220297	F6200-4138	5	32.6∗∗∗∗	116.6	5.8
13	CGF-0220228	F5854-0345	6	N/A^a^	7.5∗∗∗∗	N/A^a^
14	CGF-0220461	F6452-2137	6	70.7∗∗∗∗	93.9	67.4
15	CGF-0219869	F3243-0336	7	50.2∗∗∗∗	68.5∗∗∗∗	20.3
16	CGF-0220003	F3375-2663	8	75.8∗∗∗∗	93.7	54.8
17	CGF-0220397	F6440-4304	9	69.0∗∗∗∗	69.2∗∗∗∗	88.1
18	CGF-0220362	F6390-3434	10	51.2∗∗∗∗	81.5∗∗∗∗	121.8
19	CGF-0219785	F3202-0103	11	74.8∗∗∗∗	85.8∗∗	39.6
20	CGF-0219317	F1132-0487	11	67.3∗∗∗∗	44.4∗∗∗∗	4.4

a Due to significant toxicity, an accurate value could not be calculated.

**Fig. 4:**
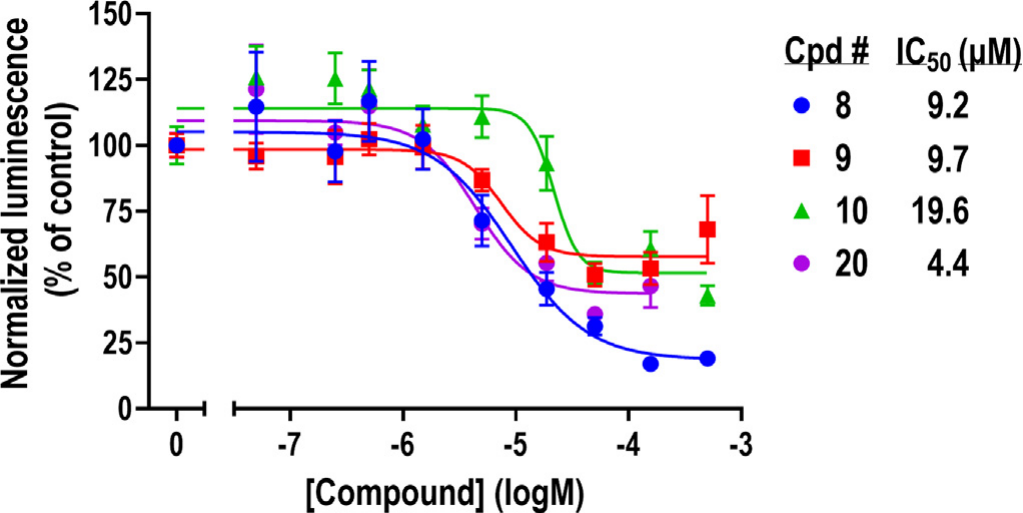
Concentration-dependent effect of hit compounds. All 20 validated hits were assayed for their ability to inhibit the RGS2-FBXO44 NanoBit signal in a concentration-dependent manner (range: 500–0.01 *μ*M). NanoBit luminescence (relative luminescence units) was normalized to viability (RFU) and presented here as percentage of untreated cells. Curves were fitted using nonlinear regression in GraphPad Prism. Representative curves of compounds **8, 9, 10,** and **20**. All compounds are shown in Supplemental Figure 5 and IC_50_ values are given in Table 1.

We next assessed the concentration-dependent effect of our 20 hits. Compounds were assayed at concentrations ranging from 500 to 0.01 *μ*M. NanoBit luminescence (relative luminescence units) was normalized to viability (RFU) and presented as a percentage of untreated cells. All compounds exhibited concentration-dependent activity (Fig. 4; Supplemental Fig. 5) with IC_50_ values as low as 4.4 *μ*M (compound **20**; all IC_50_ values presented in Table 1). With only a couple of exceptions, compounds generally displayed lower IC_50_ values than the 100 *μ*M used in our primary screen.

### 3.5 Effect of NanoBit assay hits on RGS2 protein levels

**Fig. 5:**
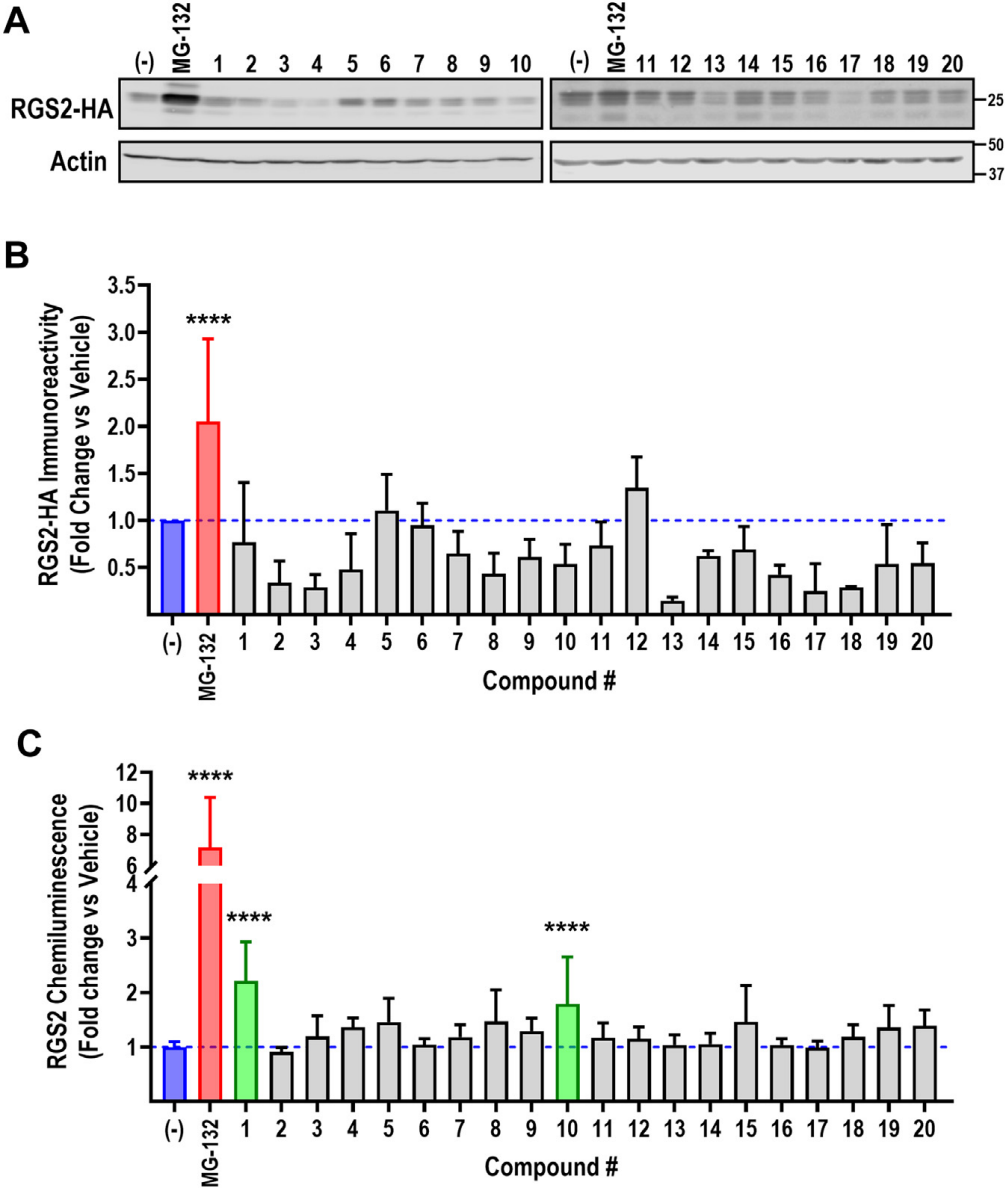
Orthogonal assay hit validation. All 20 compounds were assayed for their ability to increase RGS2 protein levels. The proteasome inhibitor MG-132 (10 *μ*M) was used as a positive control. Representative western blot (A) and quantification of 3 independent experiments (B) showing that none of the compounds significantly increase RGS2 protein levels in this assay paradigm. (C) Two of the compounds (**1** and **10**) significantly increase RGS2 protein levels in the unbiased PathHunter ProLabel assay. Due to the significant toxicity displayed by compound **1**, compound **10** was chosen for further evaluation. ∗∗∗∗*P* < .0001 compared with vehicle control using one-way ANOVA with Dunnett’s post hoc test for pairwise comparisons.

Compounds that inhibit the interaction between RGS2 and FBXO44 would protect RGS2 from proteasomal degradation and result in increased RGS2 protein levels. We therefore next assayed our 20 hits for their ability to increase RGS2 protein levels. We first performed western blot analysis of HEK-293T cells transfected with RGS2-HA and FLAG-FBXO44 and treated overnight (16–18 hours) with 100 *μ*M compound, using proteasome inhibitor MG-132 as a positive control (10 *μ*M). Using this readout, none of the compounds significantly increased RGS2 protein levels (Fig. 5, A and B). Although somewhat disappointing, given the limited sensitivity of western blot, we opted to also test the compounds using an alternate assay paradigm. Specifically, we used the PathHunter ProLabel assay (DiscoveRx), an enzyme complementation assay in which a protein of interest is tagged with a 4 kDa part of *β*-galactosidase (ProLabel; PL). The chemiluminescent signal in the assay correlates with relative levels of the PL-tagged target protein. We previously developed a stable HEK-293T-RGS2-PL cell line that was used in several high-throughput screening campaigns (Sjögren et al, 2012; Raveh et al, 2014; Sjögren et al, 2015). The PathHunter ProLabel assay offers a more quantitative measurement of protein levels, with a larger dynamic range than a western blot. We treated HEK-293T-RGS2-PL cells overnight (16–18 hours) with 100 *μ*M compound and used 10 *μ*M MG-132 as a positive control. In this assay, compounds **1** and **10** significantly increased RGS2 protein levels (Fig. 5C). Because compound **1** displayed greater cell toxicity (Table 1), compound **10** was pursued for further studies.

### 3.6 Compound **10** inhibits the RGS2-FBXO44 interaction through binding to RGS2

**Fig. 6:**
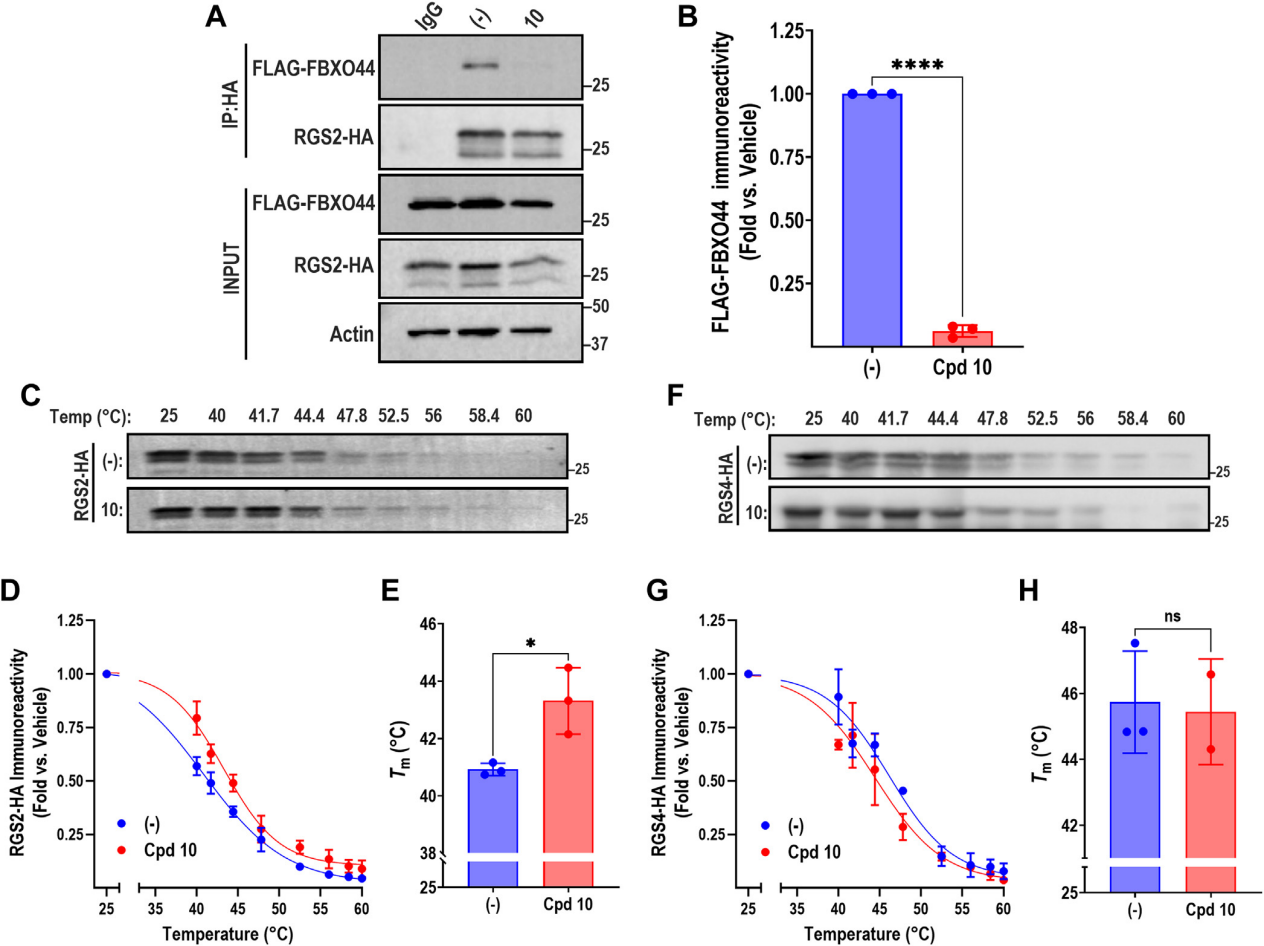
Compound **10** inhibits the interaction with FBXO44 and binds to RGS2. Representative western blot (A) and quantification of 3 independent experiments (B) demonstrating that compound **10** (100 *μ*M) significantly inhibits co-IP between RGS2-HA and FLAG-FBXO44. FLAG-FBXO44 protein levels in the IP reaction were normalized against the input levels and expressed as fold vs. vehicle (DMSO) treated cells. (C) Representative western blot of RGS2-HA from HEK-293T cells treated with 100 *μ*M compound **10** or vehicle (DMSO) and subsequent temperature exposure (40–60 °C). (D) The corresponding sigmoidal curves (mean ± SD) as determined by relative band intensity. (E) The calculated melting temperature (*T*_m_) of RGS2-HA in cells treated with compound **10**. **(**F) Representative western blot of RGS4-HA from HEK-293T cells treated with 100 *μ*M compound **10** or vehicle (DMSO) and subsequent temperature exposure (40–60 °C). (G) The corresponding sigmoidal curves (mean ± SD) as determined by relative band intensity. (H) The calculated melting temperature (*T*_m_) of RGS4-HA in cells treated with compound **10**. ∗*P* < .05; ∗∗∗*P* < .001 using Student’s unpaired *t* test.

We next used co-IP as a secondary measure to the NanoBit assay to assess if compound **10** can inhibit the RGS2-FBXO44 interaction. HEK-293T cells were transfected with RGS2-HA and FLAG-FBXO44 and treated with 100 *μ*M compound **10** overnight (16–18 hours). The cells were then subjected to co-IP using an anti-HA antibody. Compound **10** almost completely blocked the association between RGS2 and FBXO44, confirming that this compound indeed inhibits the interaction (Fig. 6, A and B).

We next assessed whether the action of compound **10** to inhibit the RGS2-FBXO44 interaction is through direct binding to either protein. Because both full-length RGS2 and FBXO44 are extremely difficult to express as recombinant proteins, we opted for a cell-based method to measure small-molecule binding, the cellular thermal shift assay (Karvonen et al, 2022). HEK-293T cells were transfected with RGS2-HA and treated with 100 *μ*M compound **10** overnight (16–18 hours). Cells were divided into PCR tubes and subjected to a temperature gradient (40–60 °C). The cells were lysed and analyzed by western blot to determine melting temperatures (*T*_m_). We observed a significant increase (2.4 °C) in the *T*_m_ for RGS2 in the presence of compound **10** (Fig. 6, C–E). We also assessed the effect of compound **10** on the *T*_m_ for RGS4, which is closely related to RGS2 and also subject to proteasomal degradation, however, independent of FBXO44 (Lee et al, 2005; Sjögren et al, 2015). Under the same conditions used for RGS2, compound **10** had no significant effect on the *T*_m_ for RGS4, suggesting some degree of selectivity for this compound (Fig. 6, F–H). Altogether, these data support the hypothesis that compound **10** inhibits the RGS2-FBXO44 interaction through direct binding to RGS2.

### 3.7 Compound **10** increases RGS2 protein levels in an FBXO44-dependent manner

**Fig. 7:**
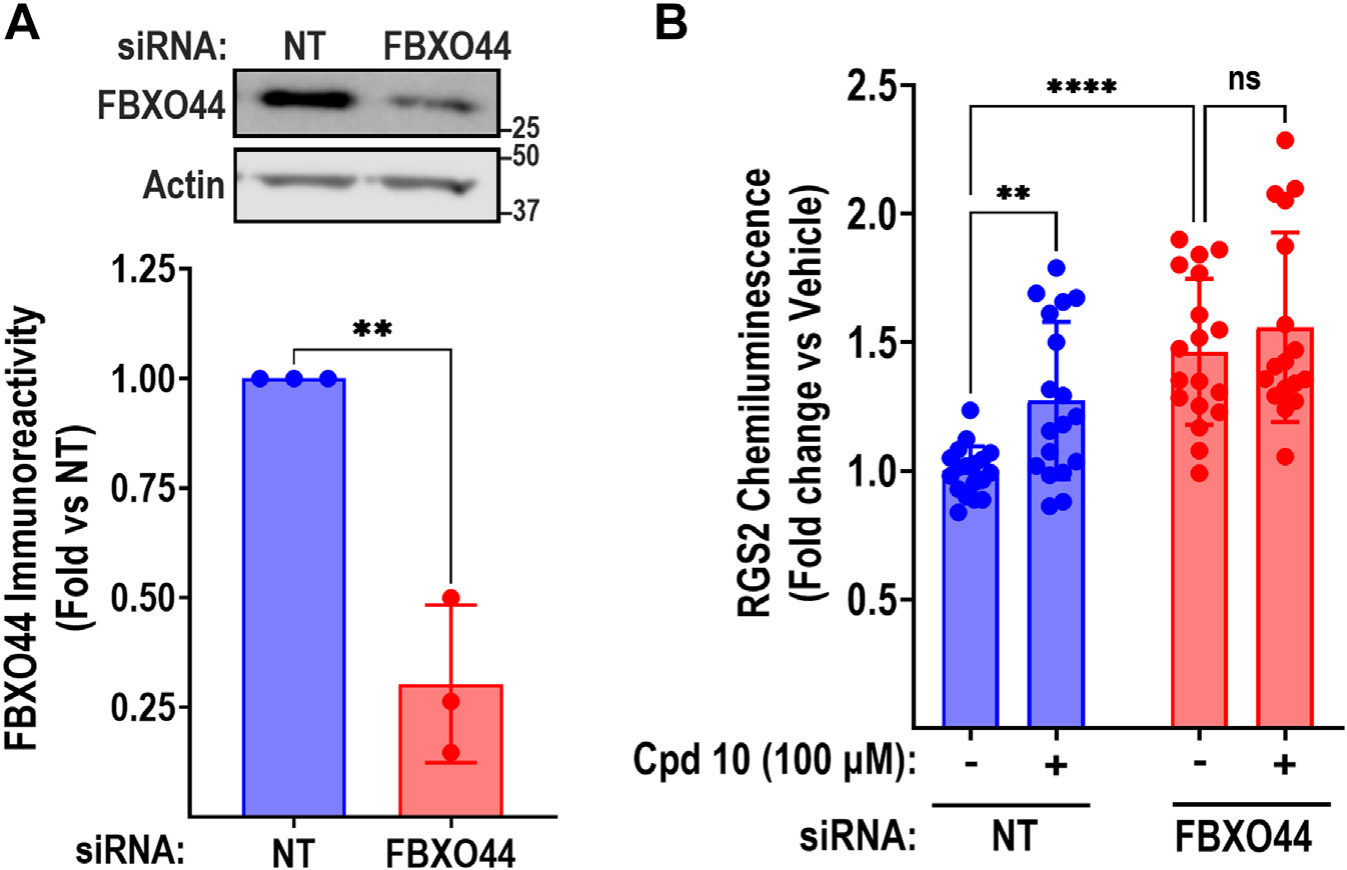
The ability of compound 10 to increase RGS2 protein levels is dependent on FBXO44. (A) Representative western blot and quantification demonstrating significant siRNA-mediated FBXO44 knockdown in HEK293T-RGS2-PL cells. ∗∗ *P* < .01 using Student’s unpaired *t* test. (B) Quantification of 3 independent experiments demonstrating that the ability of compound **10** to increase RGS2 protein levels is dependent on FBXO44, using the PathHunter ProLabel Assay. ∗∗*P* < .01; ∗∗∗∗*P* < .0001 using two-way ANOVA with Tukey’s post hoc test for pairwise comparisons.

As a further validation of the action of compound **10**, we assessed whether the ability to increase RGS2 protein levels was dependent on FBXO44 using the PathHunter ProLabel assay. HEK-293T-RGS2-PL cells transfected with either nontargeting (NT) or FBXO44-targeting siRNA were treated overnight (16–18 hours) with vehicle or compound **10** (100 *μ*M). Protein expression of FBXO44 was significantly reduced (70%; Fig. 7A) after 48 hours. RGS2 protein levels were significantly increased by either 100 *μ*M compound **10** or FBXO44 siRNA. However, siRNA-mediated FBXO44 knockdown resulted in a diminished ability of compound **10** to increase RGS2 protein levels (Fig. 7B). Thus, compound **10** increases RGS2 protein levels through a mechanism that is dependent on FBXO44.

## 4 Discussion

Targeting RGS proteins with small molecules represents a major challenge. Although some successes have been reached in developing inhibitors of the RGS-G*α* interaction (Blazer et al, 2011; Turner et al, 2012; Storaska et al, 2013; Blazer et al, 2015), enhancing RGS protein function presents an even more daunting task. Targeting mechanisms that regulate RGS protein expression, subcellular localization, and/or posttranslational modifications that affect function represent an alternative strategy. Indeed, we previously showed that increasing RGS protein levels results in augmented effects on G protein signaling in several cellular models, as well as in vivo (Sjögren et al, 2012; Raveh et al, 2014; Sjögren et al, 2016). Our subsequent identification of the CUL4B/DDB1/FBXO44 E3 ligase that targets RGS2 for degradation (Sjögren et al, 2015) provided a mechanism that can be selectively targeted to stabilize and increase RGS2 protein levels. Here, we describe the development and optimization of a high-throughput assay for screening inhibitors of the RGS2-FBXO44 interaction in live cells, and the identification of a candidate screening hit from a PPI fragment screen, which inhibits the interaction through binding to RGS2. Although many questions remain, and larger screening campaigns may be needed, this represents the first example of an RGS stabilizing tool compound that acts through selective inhibition of proteasomal degradation. In addition, our RGS2-FBXO44 NanoBiT assay can be further used to monitor the dynamics of the interaction under various treatment conditions, allowing us to fill knowledge gaps of the cellular mechanisms that regulate RGS2.

The UPS has been extensively studied for its druggability, and many strategies have been taken to target the proteasome itself, including the development of proteasome inhibitors for the treatment of several blood cancers (Gandolfi et al, 2017). However, not surprisingly, given the importance of the UPS for normal physiological functions, these drugs are associated with severe on target side effects. As a result, the use of proteasome inhibitors is limited to diseases for which severe adverse effects may be acceptable. In contrast, clinical indications for RGS2 include hypertension, a disease that commonly goes unnoticed, and any side effects of antihypertensive drugs would be associated with low compliance. Identifying the E3 ligase responsible for the degradation of a specific target would provide a more selective strategy, with reduced chances of adverse effects. Little is known regarding other substrates for FBXO44, apart from RGS2. The only other substrate identified to date is BRCA1, a gene commonly mutated in breast cancer (Brzovic et al, 1998, 2001; Lu et al, 2012). BRCA1 expression is reduced in up to 80% of breast cancer, even when no mutations are present (Thompson et al, 1995). Hence, a compound that stabilizes BRCA1 by preventing FBXO44-mediated degradation could be potentially beneficial. In addition, because compound **10**, identified here, appears to inhibit the RGS2-FBXO44 interaction by binding to RGS2, we predict that other FBXO44 substrates will remain unaffected.

RGS2 interacts with several proteins to mediate its functions. Apart from the canonical G*α* interaction, RGS2 also interacts with, and inhibits, certain subtypes of adenylate cyclase (ACII, V, and VI; Salim et al, 2003; Roy et al, 2006) and several GPCRs (Bernstein et al, 2004; Matsuzaki et al, 2011; Kim and Ghil, 2020), through its N-terminal region. It also interacts with the translation initiation factor eIF2B*ε*, through a stretch of residues partly spanning the RGS domain, to inhibit total protein synthesis (Nguyen et al, 2009). Depending on the nature of compound **10** binding to RGS2, 1 or more of these functions could potentially be affected. Future studies are needed to determine the binding site of compound **10** (and any future tool compounds) on RGS2 functions, beyond the effects on protein stability.

With over 600 E3 ligases identified to date, all with a specific set of substrates, the strategy undertaken here could and has been applied to many other proteins to stabilize them. Perhaps, the most well-developed E3-substrate pair described is the cell cycle master controller p53 and its E3 ligase MDM2. Several p53-MDM2 inhibitors have been developed, exemplified by Nutlin-3a, with the ability to induce p53-mediated apoptosis for the treatment of blood cancers, such as leukemia (Kojima et al, 2006). Apart from RGS2, other RGS proteins could also be candidates for stabilization by inhibiting their degradation. Both RGS4 and 5 are rapidly degraded through the N-end rule pathway (Davydov and Varshavsky, 2000; Lee et al, 2005), and the E3 ligase involved has been defined (Lee et al, 2011). Strategies could be undertaken to identify RGS4/5 stabilizing compounds, similar to what we present here for RGS2.

Although our current study successfully identified compound **10** as an inhibitor of the RGS2-FBXO44 interaction in cells, the vast majority of our hits could not be validated in orthogonal assays to stabilize RGS2. We propose several factors that could impact this low confirmation rate. First, the compounds within this library are fragments, with a smaller size than average screening molecules. Although they have been chosen based on properties favoring PPI inhibition, their small size may not inhibit the RGS2-FBXO44 interaction to a degree that would translate to measurable RGS2 protein stabilization. Second, because our NanoBit assay measures reduction in luminescence, any compound with a mechanism that impacts Nluc luciferase or its substrate, or causes general cell toxicity, will present as a positive hit. We have partly tried to remedy this pitfall by multiplexing our assay with a cell viability assay.

For the current study, we opted for a cell-based assay to capture compounds that would inhibit the RGS2-FBXO44 NanoBit signal in the presence of potential posttranslational modifications necessary for the interaction. We also chose this strategy for the ability to identify compounds that were cell-permeable and stable in a cellular environment. It is possible that this assay paradigm impacted the ability of compounds to inhibit the interaction, either in a positive or negative manner. Nevertheless, this study provides the first example of a small molecule with the ability to inhibit targeted RGS2 protein degradation. Future development of compounds with this mechanism of action could have therapeutic potential in pathologies associated with low RGS2 protein levels, including hypertension, heart failure, and asthma.

## Conflict of interest

The authors declare no conflicts of interest.
