# Supplementary material for: Discovery of RGS2-FBXO44 interaction inhibitors using a cell-based NanoBit assay
Source: Mol Pharmacol. 2025 Mar 19;107(5):100030. doi: 10.1016/j.molpha.2025.100030 (PMC13095444; doi:10.1016/j.molpha.2025.100030)
Supplement: Supplementary Material [file mmc1.pdf]

MOLPHA-D-24-00001

SUPPLEMENTAL MATERIAL

Discovery of RGS2-FBXO44 interaction inhibitors using a cell-based NanoBit assay

*Sadikshya Aryal[2,†], Cindy Wong[1,†], Harrison J. McNabb[2], Ahmad Junaid[2], Ryan A. Altman[2] and Benita Sjögren[1,2]**

[1]*Department of Pharmaceutical Sciences, University of California, Irvine, Irvine, CA 92697*

[2]*Borch Department of Medicinal Chemistry and Molecular Pharmacology, Purdue University, West Lafayette, IN 47907*

TABLE OF CONTENTS

Supplemental Table 1. Cluster analysis of confirmed hits. 2

Supplemental Figure 1. RGS2-FBXO44 NanoBit assay optimization. 7

Supplemental Figure 2. Separation ratio across different numbers of clusters. 8

Supplemental Figure 3. Distance matrix. 8

Supplemental Figure 4. Dendrogram illustrating the arrangement of the clusters. 9

Supplemental Figure 5. Dose-response curves of compounds 1-20. 10

Supplemental Table 1. Cluster analysis of confirmed hits. Compounds confirmed to significantly inhibit RGS2-FBXO44 NanoBit signal were subjected to chemical clustering as described in *Materials & Methods*.

Cluster No.	Molecule Name	Life Chemicals ID Number	Molecular weight (g/mol)	Structure	Tanimoto Similarity
1	CGF-0219467	F2075-0462	353.47	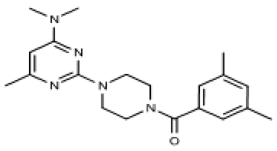	0.057
1	CGF-0219469	F2075-0493	365.481	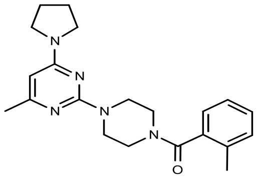	0.071
1	CGF-0059018	F2075-0495	365.481	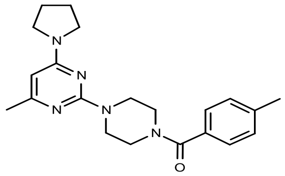	0.064
1	CGF-0059025	F2075-0589	379.508	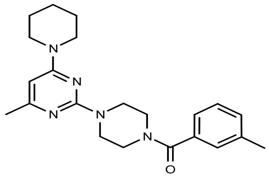	0.068
1	CGF-0220620	F6523-0550	393.414	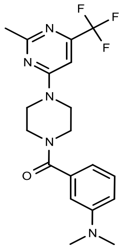	0.077
2	CGF-0220386	F6438-1673	340.427	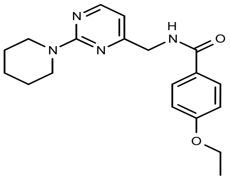	0.094
3	CGF-0219442	F2049-0352	410.514	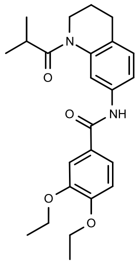	0.126

Supplemental Table 1 cont.

Cluster No.	Molecule Name	Life Chemicals ID Number	Molecular weight (g/mol)	Structure	Tanimoto Similarity
4	CGF-0219226	F0290-0255	267.39	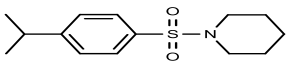	0.138
4	CGF-0053689	F0838-0022	362.28	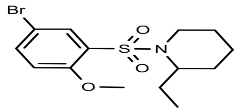	0.181
4	CGF-0219319	F1132-0541	338.24	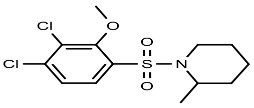	0.162
4	CGF-0219345	F1406-0127	341.47	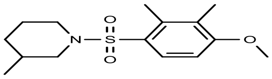	0.21
4	CGF-0219354	F1478-0152	297.41	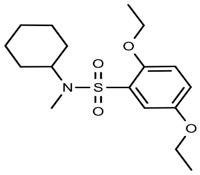	0.142
4	CGF-0219368	F1658-1239	267.39	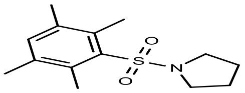	0.112
4	CGF-0219374	F1696-0075	319.84	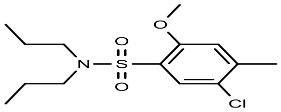	0.163
4	CGF-0219375	F1696-0101	297.41	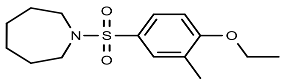	0.245
4	CGF-0219385	F1757-0299	367.51	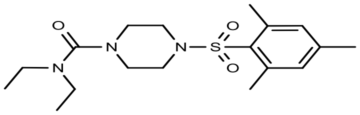	0.102

Supplemental Table 1 cont.

Cluster No.	Molecule Name	Life Chemicals ID Number	Molecular weight (g/mol)	Structure	Tanimoto Similarity
4	CGF-0219389	F1773-0447	374.5	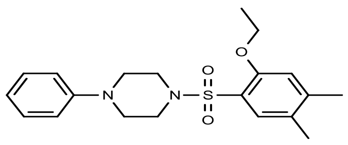	0.17
4	CGF-0219949	F3318-0665	311.44	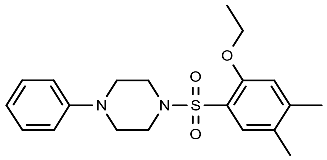	0.186
4	CGF-0220214	F5823-0948	367.89	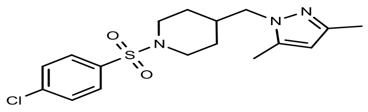	0.161
4	CGF-0220231	F5854-3982	352.42	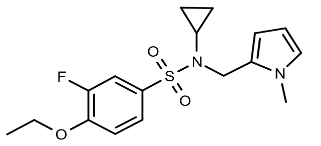	1
4	CGF-0220488	F6464-0286	393.52	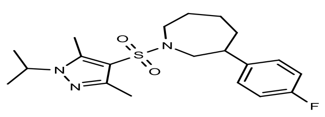	0.12
4	CGF-0220490	F6464-0765	313.84	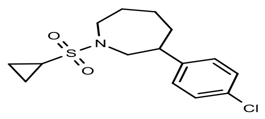	0.113
5	CGF-0220297	F6200-4138	385.89	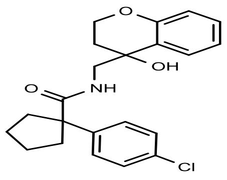	0.087

Supplemental Table 1 cont.

Cluster No.	Molecule Name	Life Chemicals ID Number	Molecular weight (g/mol)	Structure	Tanimoto Similarity
6	CGF-0220217	F5831-1946	303.42	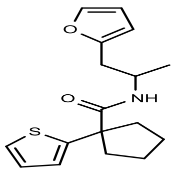	0.083
6	CGF-0220228	F5854-0345	374.528	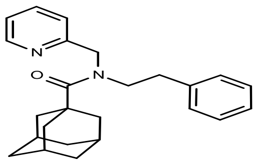	0.072
6	CGF-0220461	F6452-2137	327.468	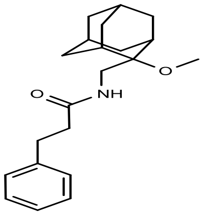	0.076
7	CGF-0219869	F3243-0336	407.558	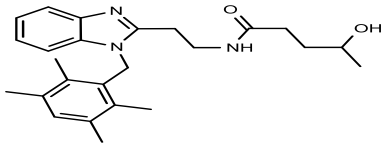	0.077
8	CGF-0220003	F3375-2663	286.379	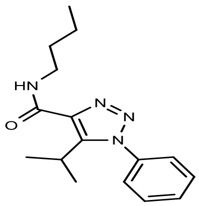	0.093
9	CGF-0220397	F6440-4304	355.5	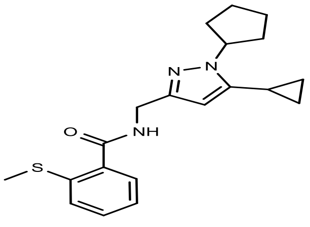	0.097
10	CGF-0220362	F6390-3434	363.9	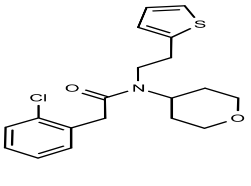	0.119

Supplemental Table 1 cont.

Cluster No.	Molecule Name	Life Chemicals ID Number	Molecular weight (g/mol)	Structure	Tanimoto Similarity
11	CGF-0219317	F1132-0487	308.4	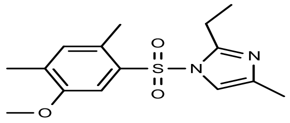	0.109
11	CGF-0219785	F3202-0103	333.83	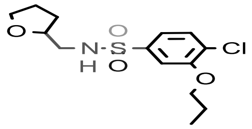	0.187
11	CGF-0219929	F3293-0263	313.41	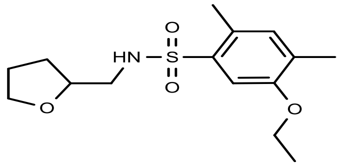	0.16
11	CGF-0220293	F6190-0782	319.84	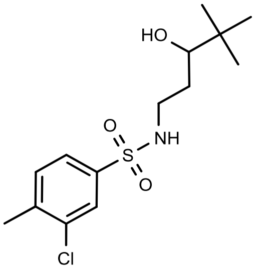	0.176

SUPPLEMENTAL FIGURES

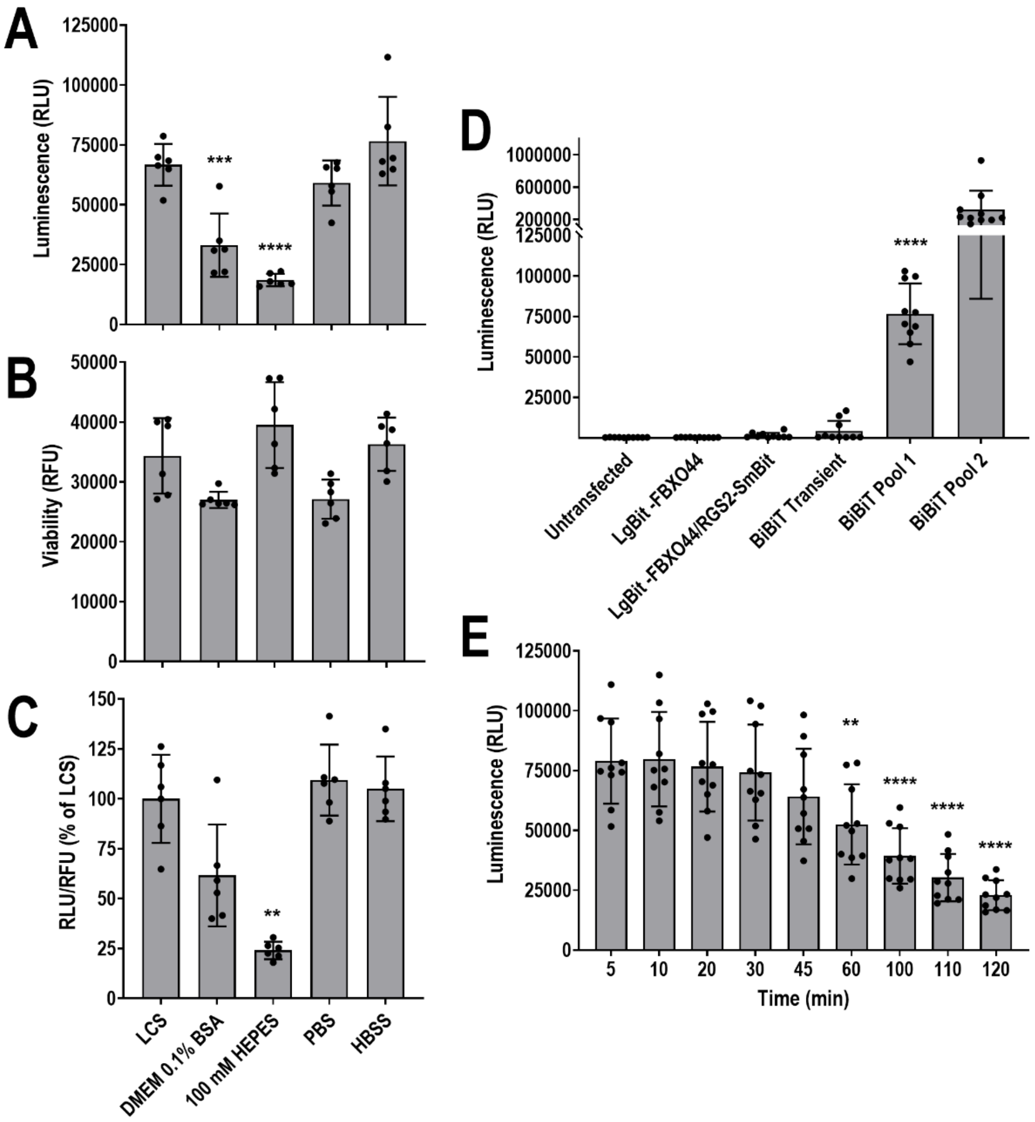

Supplemental Figure 1. RGS2-FBXO44 NanoBit assay optimization. Raw luminescence (**A**), Viability fluorescence (**B**) and normalized NanoBit responses (**C**) from buffer optimization experiments. Hank's Balanced Salt Solution (HBSS) was comparable to the supplied LCS buffer (Promega) and was chosen for further experiments. **D.** Raw luminescent signal of BiBit Pool 1 and 2 compared to HEK-293T cells transiently transfected with LgBiT-FBXO44 alone or in combination with RGS2-SmBit, as well as transiently expressed BiBiT, and mock transfected cells. While Pool 2 displays a much higher signal, it also had significantly higher variability. **E.** Luminescent signal for Pool 1 at 5-120 min after adding luminescent substrate at a cell density of 15,000 cells/well. *P<0.05; **P<0.01; ***P<0.001; ****P<0.0001 using one-way ANOVA with Dunnett's *post hoc* test for pairwise comparisons.

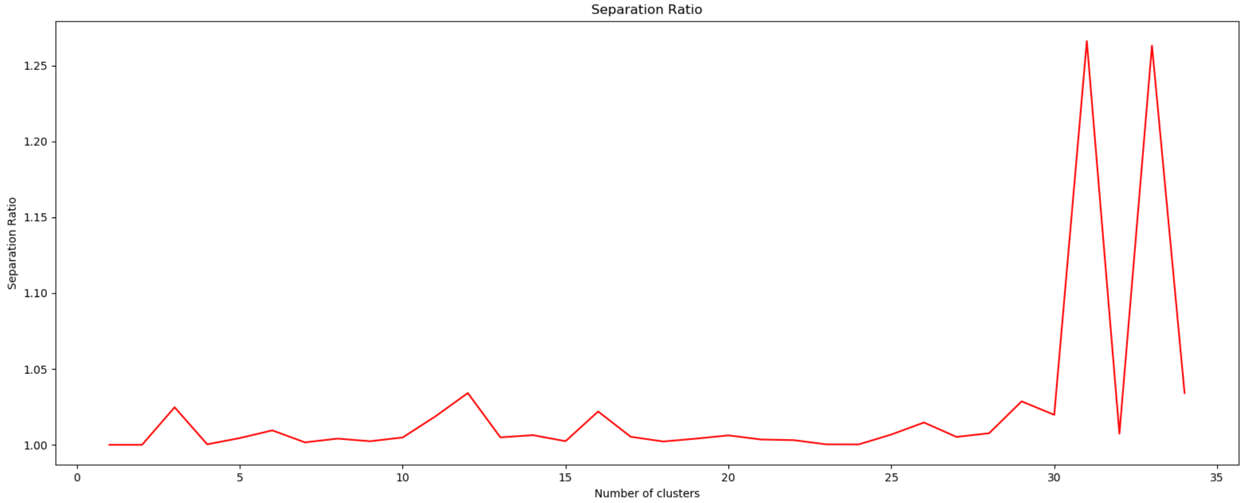

Supplemental Figure 2. Separation ratio across different numbers of clusters. The general distinctiveness of clusters remains stable across various cluster counts. However, there are specific points for which the distinctiveness significantly improves.

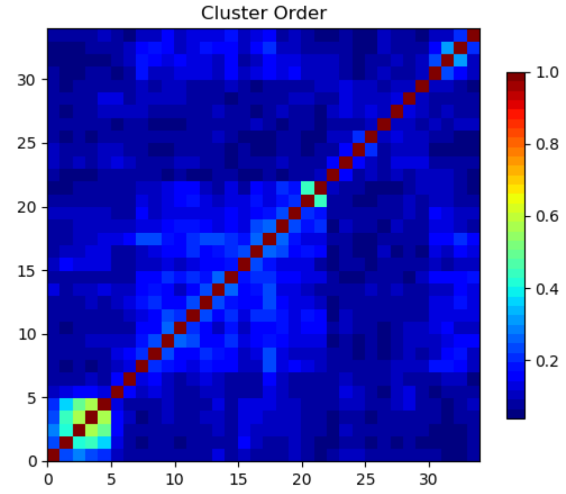

Supplemental Figure 3. Distance matrix. After reordering the data points based on clustering results, distinct blocks of higher similarity (red and yellow) become apparent. These blocks along the diagonal indicate that the clustering algorithm successfully grouped similar data points together, making the clusters more visible and distinct.

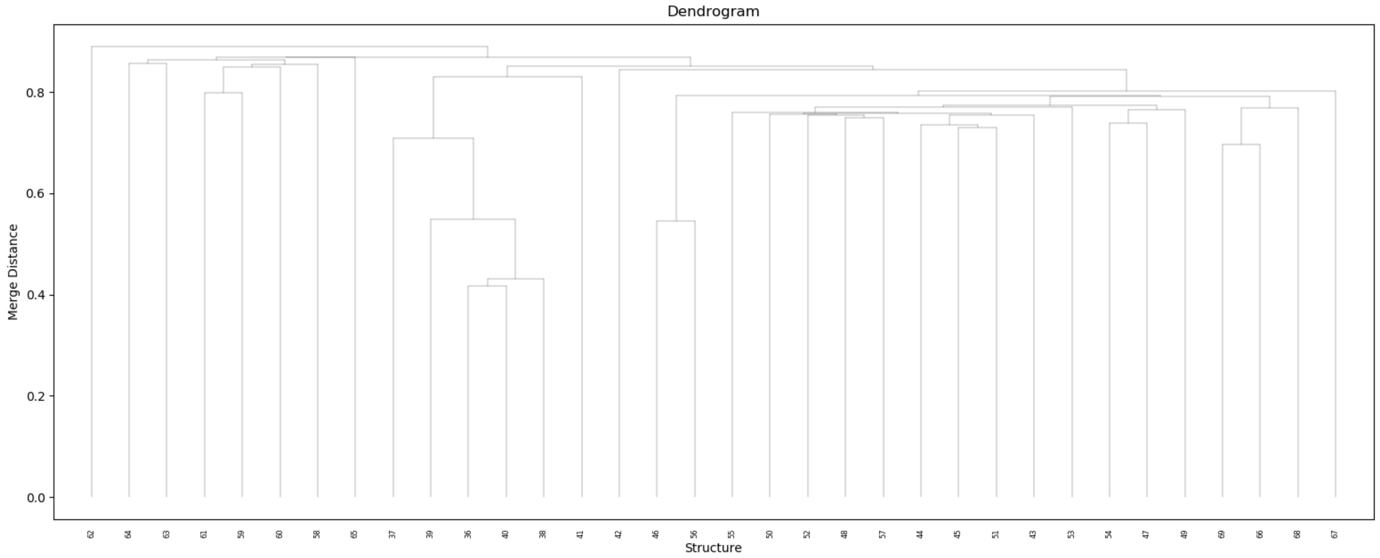

Supplemental Figure 4. Dendrogram illustrating the arrangement of the clusters. The X-axis represents the individual data points or structures being clustered. Each label corresponds to a specific data point in the dataset. The Y-axis represents the merge distance, which is a measure of dissimilarity between clusters. The higher the merge distance, the more dissimilar the clusters being joined. The dendrogram shows distinct clustering patterns, with some clusters being formed at very low merge distances, indicating high similarity among those data points.

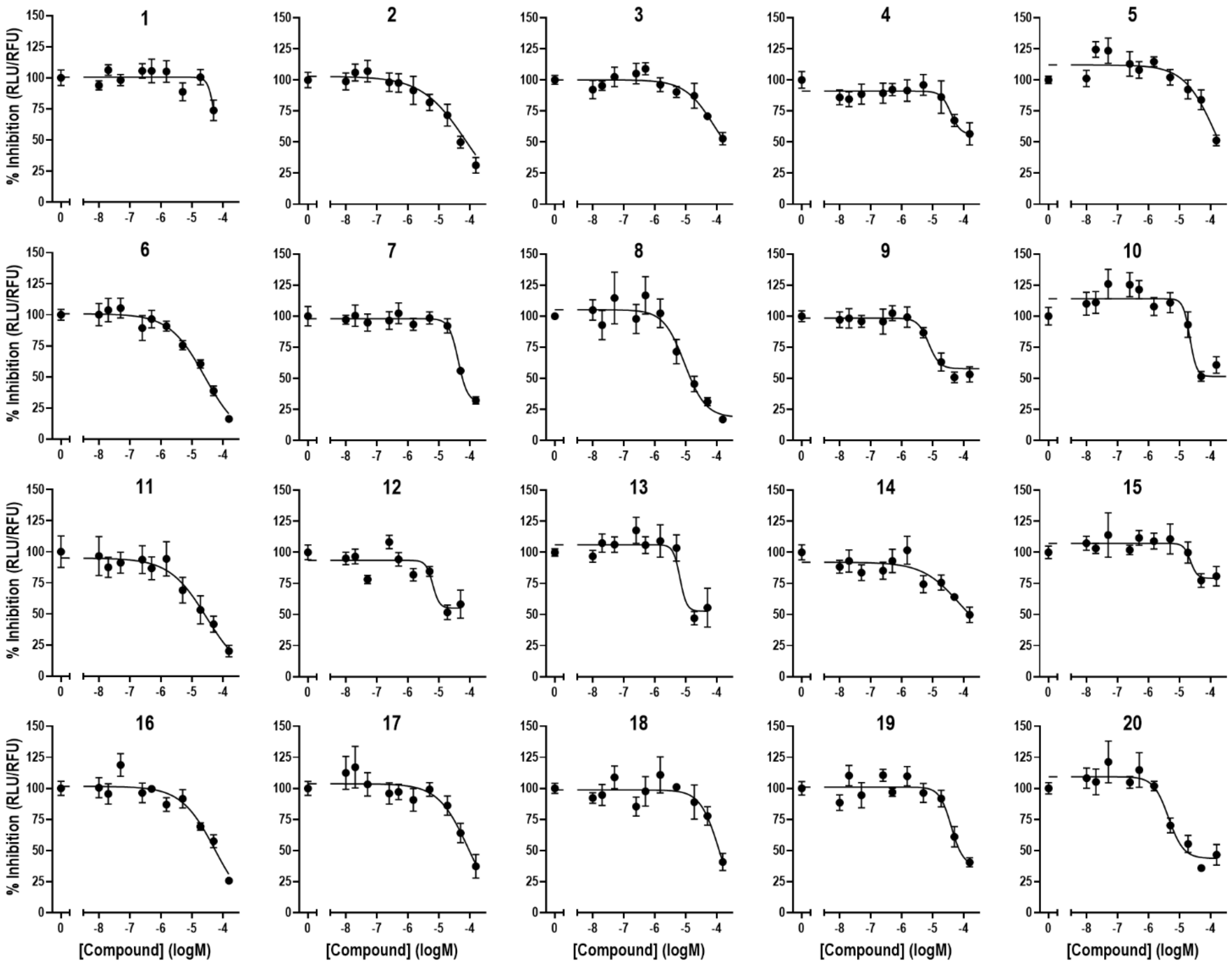

Supplemental Figure 5. Dose-response curves of compounds 1-20. Compounds were assayed at concentrations ranging from 500-0.01 μM. NanoBit luminescence (RLU) was normalized to viability (RFU) and presented here as % of untreated cells. IC_{50} values are presented in **Table 1**.
